# Enhancing Aesthetic Harmony: Comprehensive Anthropometric Lip Measurements in Youthful and Attractive Indonesian Adults for Precision Cosmetic Procedures

**DOI:** 10.1016/j.jpra.2023.12.014

**Published:** 2023-12-24

**Authors:** Lisa Y. Hasibuan, Arif Tri Prasetyo, Graciella Novian Triana Wahjoe Pramono

**Affiliations:** Division of Plastic Reconstructive and Aesthetic Surgery, Universitas Padjadjaran/ Dr. Hasan Sadikin General Hospital, Bandung, West Java, Indonesia

**Keywords:** Facial proportions, Beauty, Lip, Anthropometrics, Indonesia

## Abstract

Lips are an important part of our perception of beauty, youthfulness and attractiveness. Aesthetic lips, as with the rest of the face, differs according to age, ethnicity and sex. The aim of this study was to evaluate the anthropometric measurements of the lips of young and attractive Indonesian adults.

Photographs of faces were taken at an anterior neutral position for 100 participants; 47 men and 53 women volunteered to participate in this study. Seven landmarks were used in this study: stomion (st), sublabiale (sl), subnasale (sn), labiale superius (ls), labiale inferius (li), crista philtre (cp) and chelion (ch). Using these landmarks, lower lip height, upper lip height, philtrum length, upper vermillion height, lower vermillion height, cutaneous lower lip height, philtrum width and mouth width were measured.

The results were analysed using independent *t*-test and Mann-Whitney test. Significant differences in lip size were observed between men and women in all the measurements st-sl (lower lip height), sn-st (upper lip height), sn-ls (philtrum length), li-sl (cutaneous lower lip height), sn-ls/ls-st (philtrum length and upper vermillion height) and ls-li/ch-ch (cutaneous lower lip height and mouth width) with a significance of *p*=0.003, *p*=0.007, *p*<0.001, *p*=0.05, *p*=0.005 and *p*=0.021, respectively. Male lip measurements of ch-ch (overall lip width) and ls-st/li-st (overall lower lip height) were significantly smaller than those of female lips.

The lip ratios calculated in this study were congruent with aesthetic parameters reported in other populations. This study suggests that the same measurement standards cannot be used on different populations, but these ratios may offer a better framework for precision cosmetic procedures. We believe that the results obtained in this study on lip anthropometry will help in optimising the standard values that can be used for the Indonesian population aged 20 to 35 years.

## Introduction

The lips play an important part in our perception of facial beauty and attractiveness.^1^ Positioned at the centre of the face, the lips provide an aesthetic and youthful appearance. Lip augmentation procedures have increased in popularity, with debatable definitions of ‘ideal’ lips dimensions.^2^ Multiple factors influence the definition of ‘ideal lips’ such as age, ethnicity, culture, fashion and current trends.^3^^,^^4^ Primary aspects of lip attractiveness include the lip size, shape, fullness and symmetry. In general, the ‘ideal lip’ is full with well-defined vermillion borders and a balance between the upper and lower lips.^5^

Normal aesthetic characteristics of the mouth and lips may be useful in several medical fields. The ‘normal’ and ‘ideal’ dimensions may be used as measurements to correct craniofacial deformities such as cleft lips and malocclusion.^6^^,^^7^ Moreover, age-, sex- and ethnicity-specific databases may assist in the identification of individuals in fields such as forensics and legal medicine.

Many medical studies have evaluated changes after lip augmentation using a surgeons’ discretion rather than an objective analysis. It is essential to evaluate the average aesthetic anthropometric measurements of lips in different ethnic groups and ages. Data on lip anthropometric studies of the Indonesian population are yet to be reported. In this study, the authors aimed to evaluate the anthropometric lip measurements of young and attractive Indonesian adults.

## Methods

The volunteers were healthy, young and attractive Indonesian adults from the medical faculty of the University of Padjadjaran, Bandung, Indonesia. Written consent was obtained from all volunteers included in the study. Individuals with orthodontic or orthognathic interventions, abnormalities, malformations, deformities, surgical scars or augmentation around the lips were excluded. One hundred adults aged 20–35 years were selected for this study. Photos were taken at a natural head position with closed lips in a standardised and reproducible orientation using Nikon DSLR 5500, and all participants stood at a distance of 1 m from the camera. A ruler was placed beside the face of the participant to ensure calibration. The photographic records were analysed using the ImageJ 1.50b (Version 1.53t) software by a single investigator.

**Figure 1:**
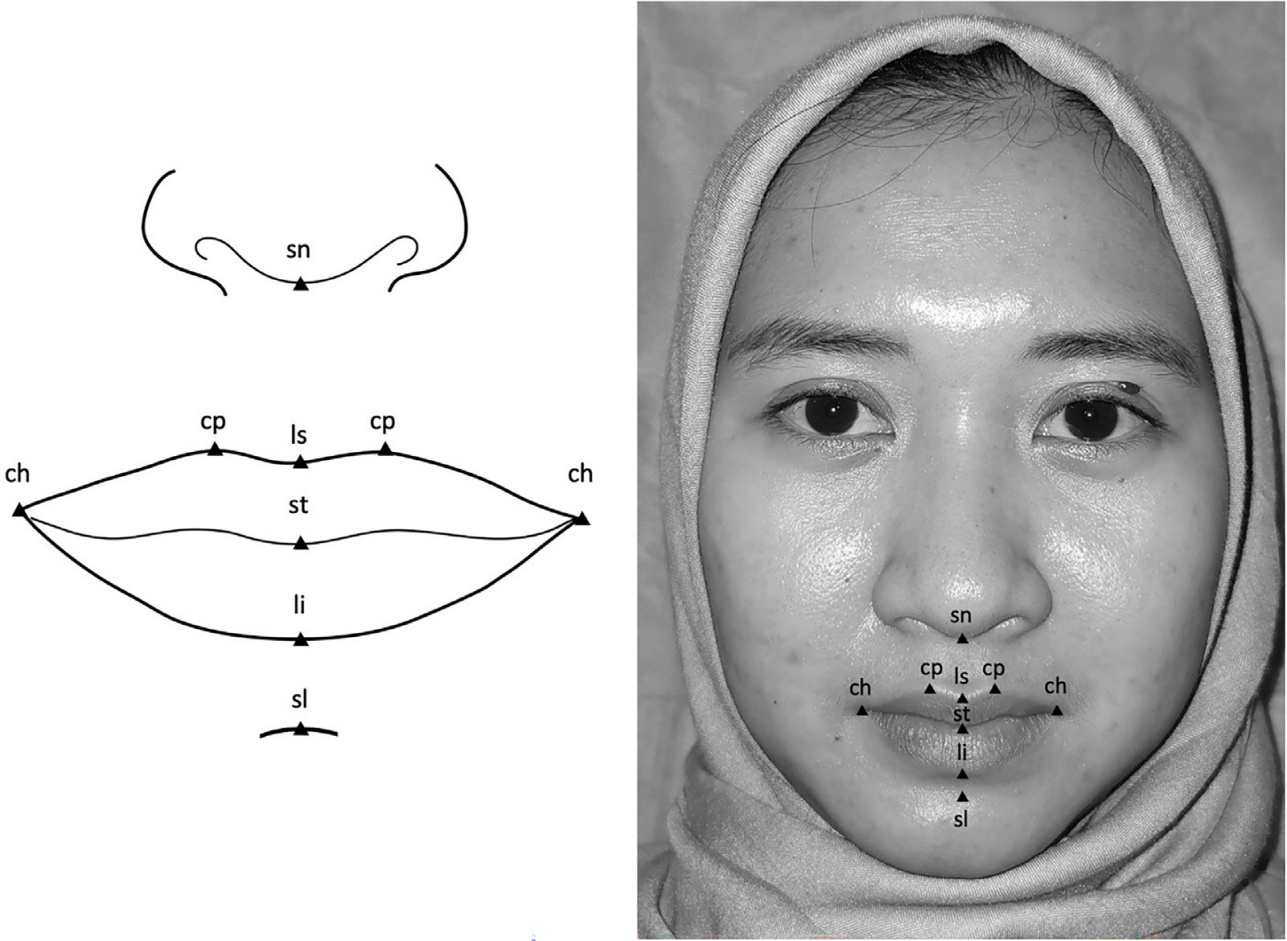
An example of the photographs and landmarks identified in this study.

Seven landmarks were identified in the photographs (Figure 1):

1. Stomion (st): The midpoint of the labial fissure.
2. Sublabiale (sl): The midpoint of the mentolabial sulcus.
3. Subnasale (sn): The midpoint at the base of the columella.
4. Labiale superius (ls): The midpoint of the vermillion border of the upper lip.
5. Labiale inferius (li): The midpoint of the vermillion border of the lower lip.
6. Crista philtre (cp): The point on the crest of the philtrum, i.e. the vertical groove in the median portion of the upper lip just above the vermillion border.
7. Chelion (ch): The point at which the outer ends of the upper and lower lip meet.

**Figure 2:**
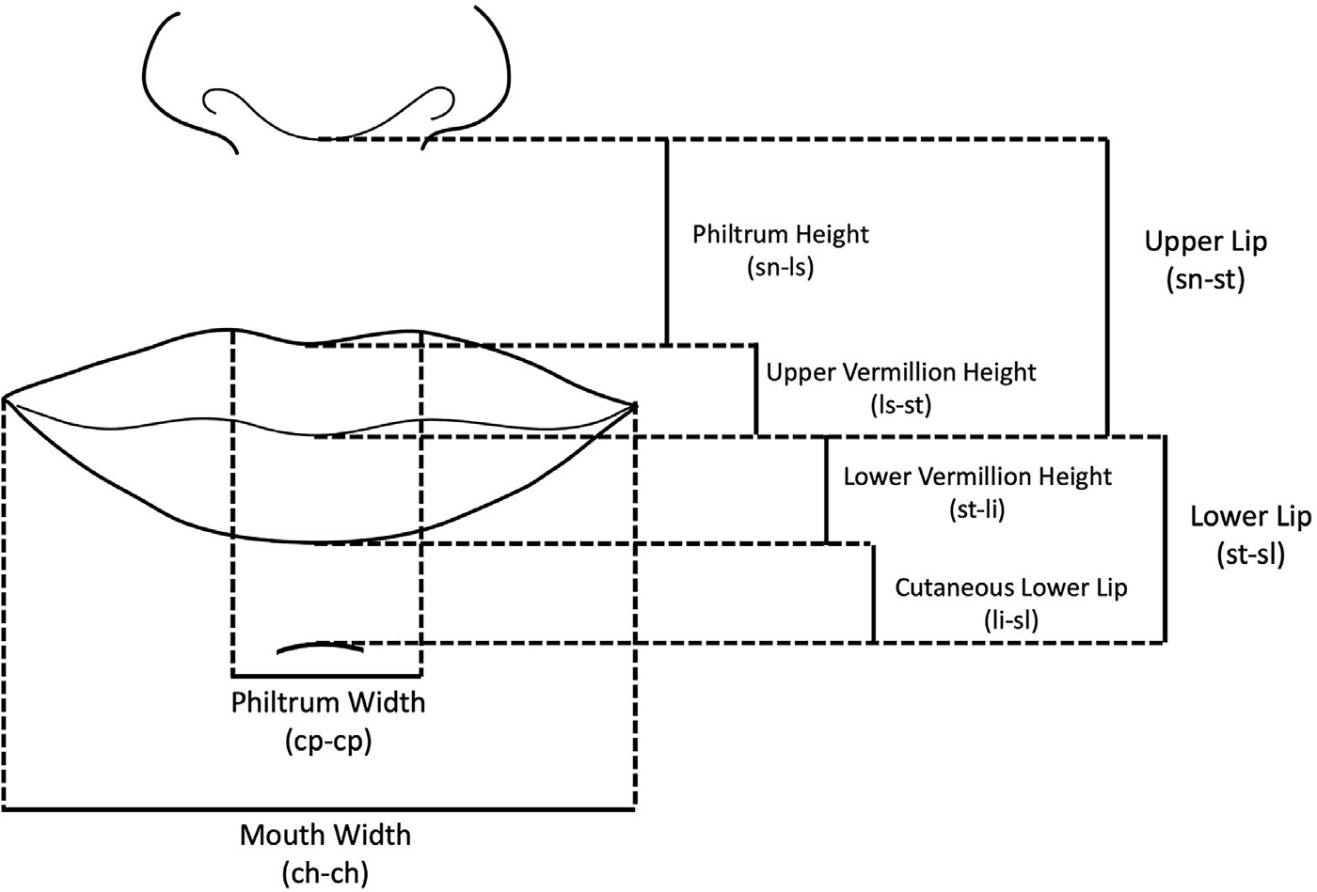
Structures measured in this study using reference landmarks.

Using these landmarks, the following lengths were measured: lower lip height (st-sl), upper lip height (sn-st), philtrum length (sn-ls), upper vermillion height (ls-st), lower vermillion height (li-st), cutaneous lower lip height (li-sl), philtrum width (cp-cp) and mouth width (ch-ch) (Figure 2).

After analysing the lengths, the following ratios were calculated.

1. ls-st/li-st: The ratio of the upper vermillion height (ls-st) to the lower vermillion height (li-st).
2. sn-ls/ls-st: The ratio of the philtrum length (sn-ls) to the upper vermillion height (ls-st).
3. li-sl/li-st: The ratio of the cutaneous lower lip height (li-sl) to the lower vermillion height (li-st).
4. sn-st/st-sl: The ratio of the upper lip height (sn-st) to the lower lip height (st-sl).
5. cp-cp/ch-ch: The ratio of the philtrum width (cp-cp) to the mouth width (ch-ch).
6. ls-li/ch-ch: The ratio of the upper vermillion height (ls-st) and lower lip height (st-sl) to the mouth width (ch-ch).

The normal distribution of data was tested using Kolmogorov–Smirnov test, and Mann–Whitney tests and independent sample *t*-test were used to compare the variables in two independent groups. SPSS 22.0 software was used for analysis, and *p*<0.05 was considered statistically significant.

## Results

There were 100 participants in this study, with 47 men and 53 women (Table 1). The average age of women was 27.47 ± 4.76 years, whereas the average age of men was 30.13 ± 3.35 years. Statistically significant differences were found between the genders (*p*=0.007).

**Table 1:** Demographic characteristics of the study participants.

	Gender		*P value*
	Male *n*=47	Female *n*=53	
**Age**			**0.007**
*Mean (SD)*	30.13 (3.35)	27.47 (4.76)	
*Mean (SD)*	28.72 (4.35)		
*Median (Min – Max)*	30 (20 – 35)		

Linear measurements of the features of the upper and lower lip were recorded (Table 2). The average upper lip height (sn-st) was 22.40 ± 2.84 mm in men and 20.78 ± 2.96 mm in women, with a significant difference between the genders (*p*=0.007). The lower lip height (st-sl) was 16.44 ± 1.33 mm in men and 15.71 ± 1.10 mm in women, with a significant difference between the genders (*p*=0.003). Average philtrum height (sn-ls) was 15.85 ± 2.67 mm in men and 13.86 ± 2.32 mm in women, with a significant difference between the genders (*p*<0.001). The upper vermillion height (ls-st) in men was 5.60 ± 1.60 mm and 6.59 ± 1.39 mm in women. There was no significant difference between the genders in terms of upper vermillion height (*p*=0.631). The lower vermillion height was 10.51 ± 2.09 mm in men and 10.02 ± 1.38 mm in women, with no significant difference between the two genders (*p*=0.564). The cutaneous lower lip height (li-sl) was 6.14 ± 1.17 mm for men and 5.69 ± 1.12 mm for women, with a significant difference between the two genders (*p*=0.050). The philtrum width was 14.50 ± 2.31 mm for men and 14.19 ± 2.23 mm for women, with no significant difference between the genders (*p*=0.502). The mouth width was 48.17 ± 6.26 mm for men and 49.24 ± 6.06 mm for women, with no significant difference between the two genders (*p*=0.390). Larger dimensions were generally found in the men for all the parameters except upper vermillion height and mouth width. Statistically significant differences were found in upper lip height (sn-st), lower lip height (st-sl), philtrum length (sn-ls) and cutaneous lower lip height (li-sl).

**Table 2:** Lip measurement results of photographic analysis.

Measurement	Gender		*P value*
	Male *n*=47	Female *n*=53	
***Lower lip height (st-sl)***			0.003*
*Mean (SD)*	16.44 ± 1.33	15.71 ± 1.10	
*Range*	(13.20 – 18.70)	(13.80 – 17.80)	
***Upper lip height (sn-st)***			0.007*
*Mean (SD)*	22.40 ± 2.84	20.78 ± 2.96	
*Range*	(16.20 – 28.90)	(14.80 – 27.50)	
***Philtrum length (sn-ls)***			<0.001*
*Mean (SD)*	15.85 ± 2.67	13.86 ± 2.32	
*Range*	(10.00 – 20.70)	(8.30 – 17.60)	
***Upper vermillion height (ls-st)***			0.631⁎⁎
*Mean (SD)*	5.60 ± 1.60	6.59 ± 1.39	
*Range*	(4.70 – 10.90)	(6.30 – 10.00)	
***Cutaneous lower lip (li-sl)***			0.050*
*Mean (SD)*	6.14 ± 1.17	5.69 ± 1.12	
*Range*	(6.40 – 8.40)	(5.50 – 8.00)	
***Lower vermillion height (li-st)***			0.564⁎⁎
*Mean (SD)*	10.51 ± 2.09	10.02 ± 1.38	
*Range*	(7.20 – 15.40)	(6.90 – 13.00)	
***Philtrum width (cp-cp)***			0.502*
*Mean (SD)*	14.50 ± 2.31	14.19 ± 2.23	
*Range*	(9.50 – 20.60)	(10.00 – 21.40)	
***Mouth width (ch-ch)***			0.390*
*Mean (SD)*	48.17 ± 6.26	49.24 ± 6.06	
*Range*	(33.60 – 59.20)	(38.20 – 61.50)	

⁎ Independent Sample t Test

⁎⁎ Mann Whitney Test

The relationship ratios between the horizontal and vertical measurements are described in Table 3. The ratio of upper vermillion height to the lower vermillion height (ls-st/li-st) was 0.64 ± 0.14 in men and 0.67 ± 0.17 in women, with no significant difference between the genders (*p*=0.516). The ratio of philtrum height to upper vermillion height (sn-ls/ls-st) was 2.53 ± 0.72 in men and 2.18 ± 0.17 in women. There was significant difference between the two genders (*p*=0.005). The ratio of cutaneous lower lip height to lower vermillion height (li-sl/li-st) was 0.61 ± 0.19 in men and 0.58 ± 0.16 in women, with no significant difference between the genders. The ratio of upper lip height to lower lip height (sn-st/st-sl) was 1.36 ± 0.16 in men and 1.32 ± 0.19 in women, with no significant difference between the genders. The ratio of philtrum width to mouth width (cp-cp/ch-ch) was 0.30 ± 0.03 in men and 0.29 ± 0.04 in women, with no significant difference. The ratio of vermillion height to mouth width (ls-li/ch-ch) was 0.36 ± 0.05 in men and 0.34 ± 0.05 in women. There was a significant difference between the genders (*p*=0.105).

**Table 3:** Relationship ratios between the horizontal and vertical measurements.

Measurement		Gender		*P value*
		Male *n*=47	Female *n*=53	
***ls-st/li-st***				0.516⁎⁎
*Mean (SD)*	Ratio of upper vermillion height to lower vermillion height	0.64 ± 0.14	0.67 ± 0.17	
***sn-ls/ls-st***				0.005*
*Mean (SD)*	Ratio of the philtrum length to the upper vermillion height	2.53 ± 0.72	2.18 ± 0.50	
***li-sl/li-st***				0.386*
*Mean (SD)*	Ratio of the cutaneous lower lip height to lower vermillion height	0.61 ± 0.19	0.58 ± 0.16	
***sn-st/st-sl***				0.296*
*Mean (SD)*	Ratio of upper lip height to the lower lip height	1.36 ± 0.16	1.32 ± 0.19	
***cp-cp/ch-ch***				0.102*
*Mean (SD)*	Ratio of philtrum width to the mouth width	0.30 ± 0.03	0.29 ± 0.04	
***ls-li/ch-ch***				0.105*
*Mean (SD)*	Ratio of upper vermillion height and lower lip height to mouth width	0.36 ± 0.05	0.34 ± 0.05	

⁎ Independent Sample t Test.

⁎⁎ Mann Whitney Test.

## Discussion

Often, an attractive face is associated with good health, prosperity and social skills.^8^ Owing to the importance of the appearance of the lips, several patients seek enhancement via surgical or non-surgical methods. However, aesthetic standards are often subjective and non-quantifiable.^9^ The definition of beauty may vary according to race, ethnicity, current trends and age.

The ‘normal’ and ‘healthy’ morphometry of the lips is important for planning lip reconstruction in patients, performing orthognathic surgery, evaluating surgical results and inclusion in forensic databases of specific ethnicities.^6^^,^^10^ The parameters measured in this study have been examined in many different ethnicities, such as Turkish,^1^ Korean,^8^^,^^11^^,^^12^ Malaysian,^13^^,^^14^ Japanese,^15^ Persian,^16^ Iranian,^17^ Indian,^18^ African-American,^19^ Anatolian^2^ and Caucasian.^20^

In this study, we observed various anthropometric measurements of Indonesian youth and reported mean results from men and women. In general, most measurements were greater in men than in women, except for those of upper vermillion height and mouth width. However, the differences in these measurements were not statistically significant between the genders. The vermillion height was 5.60 ± 1.60 mm in men and 6.59 ± 1.39 mm in women. In a study on Japanese youth, lip measurements were mostly larger in men, except for vermillion height, which tended to be approximately the same. This tendency made the vermillion look thicker in women, which is an important gender difference.^15^ Furthermore, the width of the lips was 48.17 ± 6.26 mm in men and 49.24 ± 6.06 mm in women. This is an important feature because a reduced mouth width greatly affects the aesthetics of the lower third of the face.^21^ This finding was also observed in the study by Lullari et al.^1^ Almost all the linear measurements were statistically significant and larger in men than in women, including the upper lip height (sn-st), lower lip height (st-sl), philtrum length (sn-ls) and cutaneous lower lip height (li-sl) (*p*<0,05). The data from this study are consistent with those reported by previous studies.

Ratios are beneficial for anthropometric measurements, especially to quantify aesthetic measurements. Ratios may be more beneficial considering the variations among races as well as ages.^1^^,^^22^ Chong et al. stated that lip width increased significantly with age without any change in the philtrum width. Ageing also decreases vermillion height and increases cutaneous height.^22^ Scott et al. described that a higher ratio of upper vermillion height and a lower ratio of lip height to mouth width are more aesthetic.^23^ Chong et al. described that in Chinese women in their twenties, the ratio was 0.4, changing to 0.3 in their sixties. Similarly in the study by Habal et al., the Turkish population showed ratios of 0.31 ± 0.16 in men and 0.29 ± 0.04 in women. We obtained similar results for the ratio of vermillion height to mouth width (ls-li/ch-ch), with 0.36 ± 0.05 in men and 0.34 ± 0.05 in women and no statistically significant difference between the genders.

Our results for ratio of upper-to-lower vermillion height were 0.64 ± 0.14 in men and 0.67 ± 0.17 in women, with no significant difference between the genders. These data are similar to those reported in a study by Jang et al. where the ratio of the upper vermillion height to the lower vermillion height (ls-st/li-st) was 0.69 ± 0.86 in the Miss Korea group, whereas the ratio was 0.73 ± 0.17 in the general population.^11^ In a study by Hwang et al. evaluating perceptions of attractive and healthy-looking lips using illustration of lip ratios, the perceived attractive ratio was 0.8 (or 4/5) upper-to-lower vermillion ratio.^9^ There is a significantly different preferred ratio between illustrations and physical images of attractive lips. This difference in ratios suggest that illustrations are probably not good references for medical procedures.

Notably, to date, we have only found one other study evaluating anthropometry of the Indonesian face. Reksodiputro et al. evaluated facial anthropometry of the Javanese female, but detailed measurements were not provided.^24^ Because no previous data on Indonesians are available for comparison, the authors compared their findings with anthropometric measurements from Malaysia, a neighbouring country, with individuals having the same Mongoloid anthropological origin. Research by Kumar and Selvi showed several similar measurements.^25^ Upper lip height was larger in the male Malay population, but were similar in the female population. Lower vermillion height and lip width were also very similar, but the biggest difference was in upper vermillion height, where Indonesian measurements were smaller for male and female populations. Measurement of upper vermillion height was 9.0 ± 0.21 mm for Malay men and 8.3 ± 0.21 mm for Malay women, where upper vermillion height was 5.60 ± 1.60 mm for Indonesian men and 6.59 ± 1.39 mm for Indonesian women.

Farkas et al. published an anthropometric study of the Caucasian population.^26^ When compared to Caucasian measurements, the philtrum length and lower vermillion height of the men were similar. Interestingly, upper lip height was larger in Indonesians, but upper vermillion height, female lower vermillion height and mouth width were smaller in the Indonesian population.

Indonesia consists of diverse societies, and the data obtained in this study correspond with Indonesia's largest ethnic groups. Most of the data in this research are from the Javanese (n=35) population, followed by the Sundanese (n=31) and Melayu (n=12) populations. Several other populations include the Balinese, Batak, Bugis, Minahasa and Minang. According to the 2010 sensus, the largest ethnic groups include the Javanese, Sundanese, Batak, Sulawesi, Madurese and Betawi.^27^

## Conclusion

There have been many studies examining lip anthropometry, with similar anatomical markings and measurements. In this study, we aimed to evaluate parameters, measurements and ratios for calibrating attractive, young lip measurements for the Indonesian population. We believe that further studies can be carried out in a broader range of ethnic groups, larger populations and age groups. We found many similarities in lip measurements with the Malaysian population as well as Caucasians. The most significant difference was in the height of the upper vermillion, although when calculated, the lip ratios in this research are congruent with the aesthetic parameters in other populations. This study suggests that the same measurement standards cannot be used for different populations, instead ratios may offer a better framework for precision cosmetic procedures. However, owing to a relatively small sample size, the results of this study may not be representative of the whole Indonesian population. A large prospective study is required to confirm anthropometric findings of this study.

## Ethical Approval & Consent

No ethical approval was obtained for this study; however, all participants provided informed consent.

## Patient Consent Statement for Photo Publication

We obtained written informed consent from individuals whose photographs are included in our manuscript. These individuals have been fully informed about the nature and purpose of their photographic inclusion in this publication and have consented to it. The identities of the individuals have been protected, and all photographs are used solely for educational and scientific purposes in accordance with ethical standards.
